# A Tablet-Based App to Support Nursing Home Staff in Delivering an Individualized Cognitive and Physical Exercise Program for Individuals With Dementia: Mixed Methods Usability Study

**DOI:** 10.2196/46480

**Published:** 2023-08-22

**Authors:** Jelena Krafft, Bettina Barisch-Fritz, Janina Krell-Roesch, Sandra Trautwein, Andrea Scharpf, Alexander Woll

**Affiliations:** 1 Institute of Sports and Sports Science Karlsruhe Institute of Technology Karlsruhe Germany

**Keywords:** dementia, individualized physical exercise, tailored exercise, physical activity, older adults, app, mobile health, mHealth, usability, mobile phone

## Abstract

**Background:**

The promotion of physical activity in individuals with dementia living in nursing homes is crucial for preserving physical and cognitive functions and the associated quality of life. Nevertheless, the implementation of physical activity programs in this setting is challenging, as the time and expertise of nursing home staff are limited. This situation was further exacerbated by the COVID-19 pandemic. Mobile health apps may be a sustainable approach to overcome these challenges in the long term. Therefore, the Individualized Cognitive and Physical Exercise-App (the InCoPE-App) was developed to support nursing home staff in delivering and implementing tailored cognitive and physical exercise training for individuals with dementia.

**Objective:**

This study aims to assess the usability of the InCoPE-App in terms of user performance and user perception in a laboratory setting using a mixed methods approach.

**Methods:**

Nursing home staff were encouraged to perform 5 basic tasks within the InCoPE-App. Their thoughts while using the app were captured by implementing a think aloud protocol. Then, participants completed the System Usability Scale questionnaire. The think aloud transcripts were qualitatively evaluated to unveil usability issues. All identified issues were rated in terms of their necessity to be fixed. Task completion (ie, success rate and time) and perceived usability were evaluated descriptively.

**Results:**

A total of 14 nursing home employees (mean age 53.7, SD 10.6 years; n=13, 93% women) participated in the study. The perceived usability of the InCoPE-App, as assessed by the System Usability Scale questionnaire, can be rated as “good.” The main usability issues concerned navigation logic and comprehensibility of app content.

**Conclusions:**

The InCoPE-App is a user-friendly app that enables nursing home staff to deliver and implement cognitive and physical exercise training for individuals with dementia in nursing homes. The InCoPE-App can be used with little training, even by people aged ≥50 years, who may have low digital literacy. To achieve sustainable use and high user satisfaction of the InCoPE-App in the long term, it should be implemented and evaluated in a field study.

## Introduction

### Background

More than 55 million people worldwide have dementia, with approximately 10 million new cases every year [1]. By 2050, the number of individuals with dementia is expected to increase to up to 150 million individuals worldwide [2,3]. As dementia is a noncurable disease, treatment possibilities to stop or slow the progression of disease-specific symptoms (eg, declining cognitive function and physical performance) are critical. In addition to pharmacological therapies, nonpharmacological approaches such as physical activity (PA) have gained increasing attention. A growing body of research has shown that PA may have a beneficial impact on cognitive and physical performance in individuals with dementia [4]. However, only small and mainly nonsignificant effects of PA on quality of life (QoL) among individuals with dementia have been reported [5,6]. Overall, results from studies are conflicting, mainly owing to heterogeneous sample sizes and characteristics and differing intervention contents, periods, frequency, and duration of PA training [4]. Some studies also pointed out the heterogeneous prerequisites of individuals with dementia such as varying interindividual degrees of cognitive and motor impairments. Thus, a *one-size-fits-all* PA approach may fall short [7]. In addition, individual vulnerabilities and needs of individuals with dementia may need to be considered when designing, planning, and conducting PA interventions [8-10].

According to several studies [11-14] and a systematic review [15], up to 80% of individuals living in nursing homes in European countries experience dementia. Individuals with dementia residing in nursing homes often have decreased life expectancy [16], more advanced dementia stages, and more impaired physical performance compared with community-dwelling individuals with dementia [17]. Moreover, living in a nursing home is associated with negative changes in QoL [18]. Overall, promoting PA in nursing home settings is therefore crucial. In many nursing homes, PA promotion is not regarded as a task or responsibility of nursing home staff and is usually delegated to external providers (eg, physiotherapists) [19]. During the COVID-19 pandemic, this practice was no longer feasible, as many nursing homes in Germany and other European countries were closed to visitors or external service providers, and PA programs had been discontinued in many nursing homes owing to increased safety measures [19]. The resulting social isolation and restricted movement possibilities led to worsening of cognitive function and physical performance among individuals with dementia, as perceived by nursing home staff [20]. Moreover, some studies reported significant impact on the mental well-being of nursing home residents (eg, QoL) [21]. A conclusion that can be drawn from the COVID-19 pandemic with its far-reaching health consequences is that PA promotion in nursing homes should be designed and implemented in a way that allows continuation even as new challenges arise (eg, changing circumstances owing to the pandemic or similar events) and without access to external PA instructors. Therefore, mobile health (mHealth) apps may be a viable solution in this context. Various definitions of the term *mHealth* exist and most include key aspects such as mobile computing, medical sensor, and communications technologies [22], health information and services [23], patient monitoring devices, and personal digital assistants [24] to improve health outcomes. mHealth can be considered as a subsection of eHealth [23]. mHealth solutions are considered to be feasible, can be implemented at little or no cost [25], and have wide reach among various patient groups or populations.

So far, a large number of mHealth apps for use in care settings are available, with most of them providing support for medication management or health information, and they can be accessed free of charge from app stores [26]. However, to the best of our knowledge, no mHealth app for individualized PA promotion in nursing homes is available so far [27]. mHealth apps are promising tools in this setting and may help alleviate nursing home staff shortages; for example, a standardized, mHealth-based training manual may facilitate the instructions of PA sessions. Moreover, such an app may contain pictures and detailed exercise descriptions and information about the possible risk factors of certain exercises. These advantages may reduce the potential barriers for nursing home employees to deliver PA programs to individuals with dementia and enable the implementation of PA even in times of a pandemic. Nevertheless, a recent Cochrane review showed that health care workers with limited experience in using mobile apps and low digital literacy had concerns about making mistakes when using a mobile device [28], which might, in turn, affect the usability and acceptability of such apps.

However, to guarantee the long-term use and acceptability of mHealth apps in nursing homes, the feasibility and usability of an app must be considered, ideally in the design and development phase of the app [29]. Usability indicates how a product is perceived by an intended user to achieve a specific goal in a specific context of use [30]. Nevertheless, most of the currently existing mHealth apps have not been scientifically designed and empirically evaluated [31,32], and publications addressing their feasibility and usability are lacking [33]. This is a main research gap, particularly because theory-based design and development of apps with subsequent scientific evaluation of usability and acceptability may be among the most important criteria to ensure the long-term implementation of mHealth apps, particularly in special settings such as nursing homes [34]. Moreover, studies have shown that involving nursing home staff in the development process of a mobile app makes them feel valuable and appreciated, which, in turn, could have a positive impact on acceptance [35]. Therefore, an iterative development process of an app including qualitative and quantitative methods to integrate possible end users in the development process is recommended [36], where designing, testing, and redesigning of a mobile app are embedded in a regular circle [29]. Examples for qualitatively collected data could be the identification of specific problems. In contrast, quantitative data may provide insight into use times or success rates [33]. A multistep development approach is intended to increase end users’ acceptability of an mHealth app and to ensure long-term use.

### Objective

To address the current need for a scientifically derived mHealth-based PA promotion for individuals with dementia in nursing homes, we developed the Individualized Cognitive and Physical Exercise-App (the InCoPE-App). The InCoPE-App is a tablet-based app aimed at assisting nursing home staff in delivering tailored cognitive and physical exercise training for individuals with dementia in a nursing home setting. The content of the InCoPE-App is based on previous studies of our research group on PA for individuals with dementia [8-10]. The goal of this study was to evaluate the usability of the InCoPE-App with possible end users, that is, nursing home staff, using a mixed methods approach in a laboratory setting. Specifically, we examined user performance and perception, existing problems, and possible solutions regarding the InCoPE-App by integrating qualitative and quantitative methods. The results of this study will be used for further improvement and adaption of the InCoPE-App with the ultimate goal of implementation and long-term use of the app in nursing homes. Furthermore, this procedure can be used as an example for future studies of app development in nursing home settings.

If and when the InCoPE-App has high usability, we anticipate that its use by nursing home staff will likely increase PA among individuals with dementia residing in nursing homes, as the app is designed such that it empowers nursing home staff to administer tailored physical exercise training to individuals with dementia in an easy and low-threshold way. Importantly, the InCoPE-App can be used by staff without previous PA-specific training or expertise.

## Methods

### Study Design and Participants

To evaluate the usability of the InCoPE-App, we used a mixed methods approach. We used a combination of qualitative and quantitative methods and considered a sample of 14 individuals, as previous studies have shown that 8 participants are sufficient to identify the main usability problems of a system [37]. Participants were recruited in April 2021 from 5 nursing homes in South-Western Germany. To be included in the study, participants (ie, nursing home staff) were required to have had previous experience with PA programs for individuals with dementia in the nursing home setting. Before the study, eligible participants received a project description regarding the objectives, participation, and benefits of the study and provided written consent for participation. The study was registered in the German National Register of Clinical Trials (DRKS00024069).

### Ethics Approval

The study was approved by the Ethics Committee of the Karlsruhe Institute of Technology (Karlsruhe, Germany).

### The InCoPE-App: Content and Development

**Figure 1:**
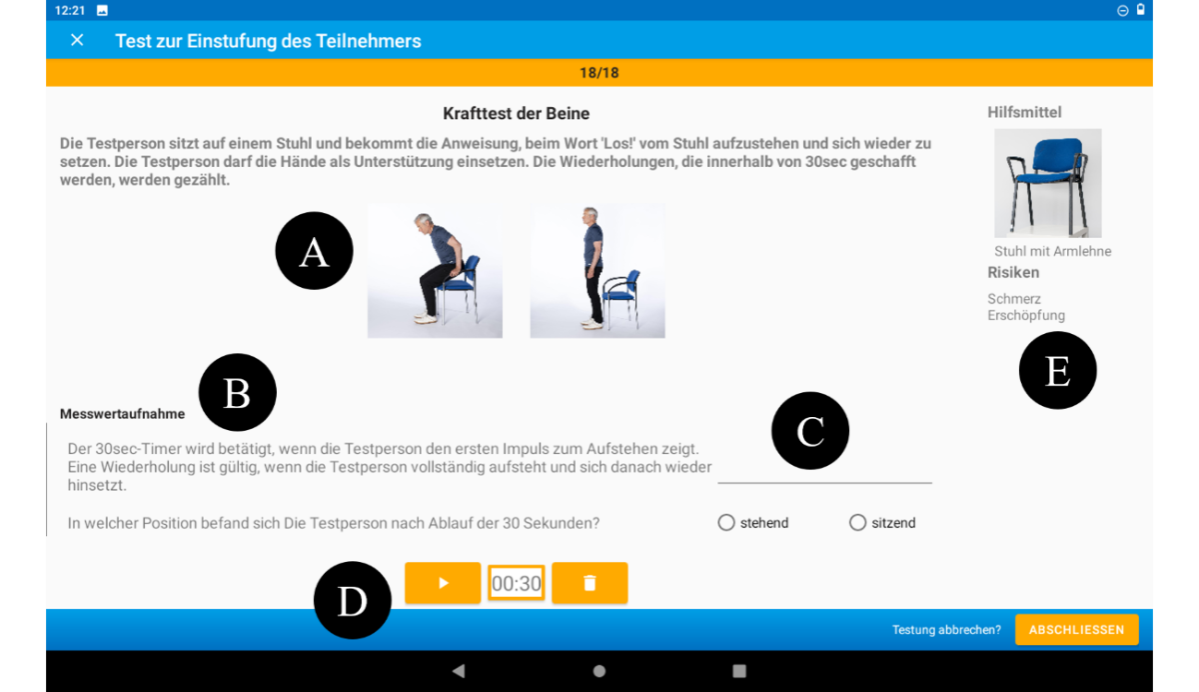
Chair stand test. (A) Written and illustrated description of the test procedure; (B) description of the measurement recording; (C) input field for the measured value; (D) integrated stop watch; and (E) required tools or equipment and possible risks.

The InCoPE-App was designed to be used by nursing home staff and not by individuals with dementia themselves, as individuals with dementia in nursing homes would not be able to perform structured physical exercise alone, and they need supervision for safety reasons. Specifically, the InCoPE-App supports nursing home staff in assessing current levels of cognitive and physical performance of individuals with dementia and, based on this assessment, guiding and delivering physical exercise sessions to individuals with dementia, without the need of having completed specific training or certification in sports or exercise science or kinesiology. A unique feature of the InCoPE-App is its integrated algorithm that uses data from 1 cognitive (ie, Mini Mental Status Examination [38]) and 3 physical performance tests (ie, Frailty and Injuries: Cooperative Studies of Intervention Techniques [39], 6-meter walk test [40], and modified 30-second chair stand test [41,42]) to tailor the recommended exercise program to the participant’s individual needs (Figure 1). The cognitive and physical tests integrated into the InCoPE-App are oriented to recommendations for individuals with dementia [43,44]. On the basis of the individual performance results, each individual with dementia is assigned to one of four exercise clusters, which are integrated in the app [45]: (1) individuals with below-average cognitive and physical performance, (2) individuals with average cognitive performance and above average physical performance, (3) individuals with above average cognitive performance and below average physical performance, and (4) individuals with above average cognitive and physical performance. The clustering into these 4 groups is based on previous studies by our group that have demonstrated the need for individualization of PA programs for individuals with dementia [8-10,46]. Depending on the cluster assignment, the InCoPE-App generates an exercise plan that fits the current performance level and needs of the individual with dementia. To adjust the exercise plan to individual changes in cognitive and physical performance, the InCoPE-App reminds the nursing home staff to repeat and record cognitive and physical performance tests every 3 weeks. In general, the exercise plan integrated into the InCoPE-App consists of ritualized warm-up and cooldown and 2 individualized workout phases that integrate exercises for balance, mobility, and upper and lower body strength [45].

**Figure 2:**
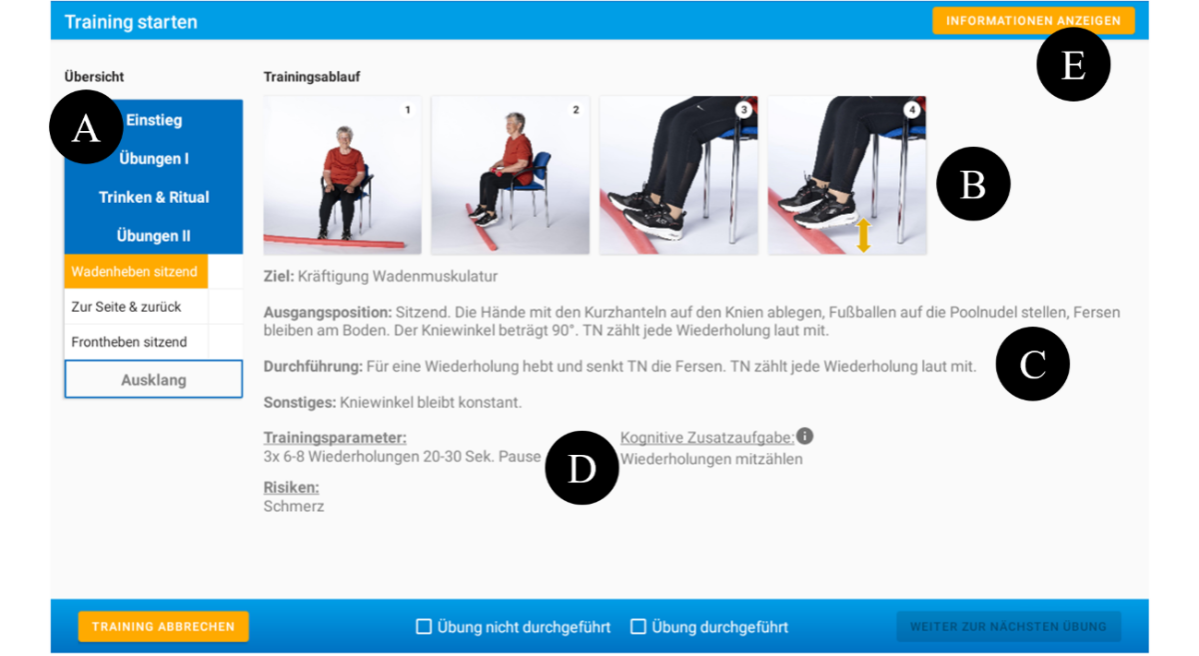
Exercise for lower limb strength. (A) Overview of the training schedule; (B) exercise sequence in pictures; (C) description of aims and correct conduct of the exercise; (D) training parameters (eg, repetitions), possible risks (eg, pain), and cognitive input (eg, counting the repetitions); and (E) further information (eg, required equipment).

The generated exercise plan is presented in the app through brief descriptions along with pictures of the exercises to provide guidance about how to perform the exercises correctly and avoid common mistakes (Figure 2). Each training session lasts 60 minutes and is intended to be performed in one-on-one sessions or small groups of up to 2 individuals with dementia. For more information about the main functions of the InCoPE-App, refer to Multimedia Appendix 1.

**Figure 3:**
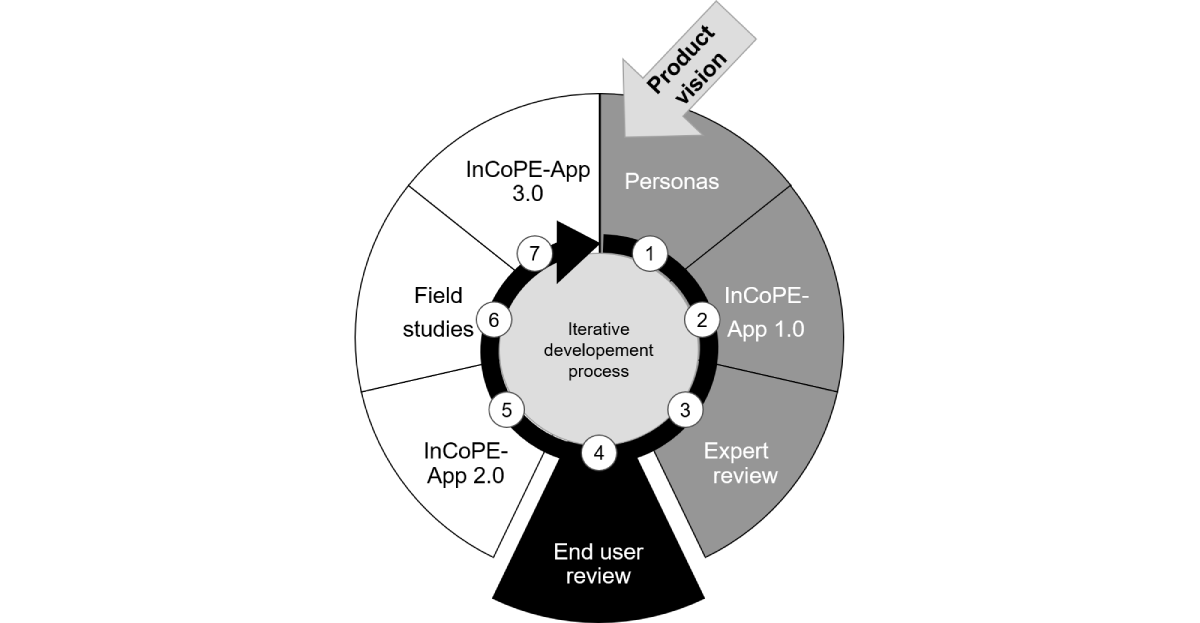
The iterative development process of the Individualized Cognitive and Physical Exercise-App (InCoPE-App) (step 1 results are published in a paper by Barisch-Fritz et al [26]; step 3 results are published in another paper by Barisch-Fritz et al [47]).

The iterative development process of the InCoPE-App included several steps (Figure 3), of which 3 are already completed. First, we defined a general *product vision* of the InCoPE-App. We then conducted a web-based survey to collect information about sex, age, profession, and daily tasks from nursing home staff. Furthermore, we gathered information about potential previous implementations of PA programs or interventions in participants’ nursing homes. On the basis of the results of this study, we were able to sketch *personas* as possible end users of the InCoPE-App [26]. In the second step, based on our product vision and the design of personas, we developed the first prototype of *the* *InCoPE-App 1.0* in collaboration with a software expert team. The InCoPE-App was developed on Android 9.0. For study purposes, an offline-capable version of the InCoPE-App was locally installed on tablets (Lenovo Tab M10; 10 inch). Currently, the app is available only in German. The usability of the InCoPE-App 1.0 was tested by 7 experts in the areas of psychology, IT, sports science, and software development using a think aloud protocol and the System Usability Scale (SUS) [47]. The *expert review* unveiled relevant information about the usability of the InCoPE-App. The experts rated the InCoPE-App as acceptable but also noted some usability problems.

### Outcomes and Procedure

After the participants signed the consent form, demographic information and data about general smartphone, tablet, and app use were collected from each participant using a short survey. Usability was assessed qualitatively and quantitatively in individual sessions during the first use of the InCoPE-App. To collect qualitative usability data, the think aloud technique was applied as it was found to be the most frequently used qualitative approach in usability testing of eHealth applications [31]. At the beginning, we explained to the participants that they would be required to speak their running thoughts aloud while interacting with the InCoPE-App. To become familiarized with this method, participants received a sample task within the InCoPE-App (ie, “Go to ‘exercise pool,’ choose exercise ‘Rope Pulling’ and tell me possible risks of this exercise”). Then, they were asked to perform 5 tasks (Table 1) with the InCoPE-App along a standardized protocol. These tasks were representative of a real-world situation when using the InCoPE-App in the nursing home setting [37]. During the think aloud session, a researcher was present and only interrupted participants if they stopped talking for >10 seconds while performing the tasks. Running thoughts of the participants were recorded via a voice recorder. Following the think aloud session, participants were asked three final questions: (1) “Which parts of the InCoPE-App are well designed?” (2) “Which parts of the InCoPE-App need to be revised?” and (3) “Do you have any other further comments on the InCoPE-App?”

For quantitative usability assessment, the time spent on each individual task and all tasks overall was assessed by using the screen recorder function of the tablet. Furthermore, the success rate of each task was coded as “success,” “problem,” or “failure,” as described by Ehrler et al [48]. After the think aloud protocol, participants completed the German version of SUS [49,50], which is one of the most frequently used questionnaires in usability research [31]. The German version of SUS has reasonable reliability (0.84), concurrent validity (0.74), and sensitivity (0.83) [50]. SUS comprises 10 statements about the usability of a system (eg, “I think that I would like to use this system frequently”), each rated on a scale ranging from “I don’t agree” to “I totally agree.” Negatively worded statements (even numbers) are coded from 4 to 0, whereas positively worded (odd numbers) statements are coded from 0 to 4 [51]. The items are added to a sum score (minimum 0; maximum 40 points), which is multiplied by 2.5 (sum score—minimum 0; maximum 100 points). Published literature suggests a mean SUS score of 68 as a useful “benchmark” [52]. Furthermore, the total SUS score can be interpreted as follows: scores <60 indicate substantial usability problems, scores between 60 and 80 indicate marginal to good usability, and scores >80 indicate good to excellent usability of a system [49]. According to the Subjective Rating Scale of Bangor et al [53], a mean SUS score of 71.4 indicates good usability.

**Table 1:** Standardized “think aloud” protocol.

Task	Description of the task
1	“Create a new test person.”
2	“Start and complete cognitive and physical testing with the test person.”
3	“Create an exercise plan and replace two exercises.”
4	“Start and finish a training session with the test person.”
5	“Start and finish a training session with two participants simultaneously.”

### Data Collection and Analysis

Each think aloud session and the 3 interview questions were recorded with a voice recorder (Philips DVT2050) and transcribed verbatim using a transcription software (software F4transkript, from audiotranskription, dr.dresing&pehl GmbH). The transcribed protocols contained time stamps to estimate the time for task completion. To identify usability problems, bottom-down developed categories (ie, navigation, screen layout, graphics, comprehensibility, and overall usability) were used to analyze the protocols divided according to the think aloud tasks. This categorization was adjusted and based on a proposal by Kushniruk and Patel [54]. Two researchers (JK and ST) coded the transcripts independently. In case of ambiguities and discrepancies, a third researcher (BBF) was consulted. The identified usability problems were further rated by 1 researcher (JK) using the Nielsen severity scale (0=I do not agree that this is a usability problem at all, 1=cosmetic problem only, 2=minor usability problem, 3=major usability problem, and 4=usability catastrophe) [55]. This allows ranking of the usability problems and helps to prioritize them for a further revision cycle of the InCoPE-App. For presentation in this paper, the quotations from the final interviews were translated from German to English.

The total SUS score, time spent on each task and in total (derived from the screen records), and frequencies of identified usability problems were evaluated descriptively (mean, SD, and range) using SPSS (version 27.0; IBM Statistics). The success rate for each task was evaluated in percentages.

## Results

### Participants

We included 14 employees (n=13, 93% women and n=1, 7% men) from 5 nursing homes. The mean age was 53.7 (SD 10.6) years. Data about general smartphone and tablet use showed that all participants (14/14, 100%) owned a smartphone, with 93% (13/14) of the participants reporting daily use. Only 21% (3/14) of the participants reported using a tablet. Of the 14 participants, 12 (86%) had several apps installed on their personal smartphones or tablets and 7 (50%) reported daily app use. For study purposes, all participants (14/14, 100%) used the InCoPE-App installed on a tablet. Participants’ demographics and information about technical experience are presented in Table 2.

**Table 2:** Sample characteristics (N=14).

Characteristics			Values
Age (years), mean (SD)			53.7 (10.6)
**Sex, n (%)**			
	Female	13 (93)	
	Male	1 (7)	
**Age group (years), n (%)**			
	20-29	1 (7)	
	30-39	1 (7)	
	40-49	1 (7)	
	50-59	6 (43)	
	>60	5 (36)	
**Certificate of secondary education, n (%)**			
	Hauptschule (diploma after 5 y)	5 (36)	
	Realschule (diploma after 6 y)	2 (14)	
	High school diploma (diploma after 8-9 y; university entrance qualification)	5 (36)	
	University degree	2 (14)	
**Use of mobile devices, n (%)**			
	Smartphone	14 (100)	
	Tablet	3 (21)	
**Frequency of smartphone use, n (%)**			
	Daily	13 (93)	
	Several times/wk	1 (7)	
	Several times/mo	—^a^	
	Rarely	—	
	Never	—	
**Frequency of tablet use, n (%)**			
	Daily	1 (7)	
	Several times/wk	2 (14)	
	Several times/mo	1 (7)	
	Rarely	—	
	Never	10 (71)	
Use of apps, n (%)			12 (86)
**Frequency of mobile app use, n (%)**			
	Daily	7 (50)	
	Several times/wk	3 (21)	
	Several times/mo	—	
	Rarely	2 (14)	
	Never	—	

^a^Not applicable.

### SUS Scores

The mean SUS score was 72.3 (SD 18.9; range 45-95), indicating good to marginal usability. According to the Adjective Rating Scale by Bangor et al [53], usability can be rated as *good*. When dividing the sample into 3 age groups (ie, nursing home staff aged <50 years: 4/14, 29%; aged between 50 and 60 years: 5/14, 36%; and aged >60 years: 5/14, 36%), the mean SUS scores were 77.5 (SD 16.2), 78 (SD 17.1), and 60 (SD 22.1), respectively, indicating better usability in participants aged <60 years. The results for single items of the SUS are presented in Table 3.

**Table 3:** Scores for the single items of the System Usability Scale.

Item	Statement	Score of the total group (N=14), mean (SD)^a^	Score of participants aged ≤50 years (n=4), mean (SD)^b^	Score of participants aged 51-59 years (n=5), mean (SD)^c^	Score of participants aged ≥60 years (n=5), mean (SD)^d^
1	“I think that I would like to use this system frequently.”	3.2 (1)	3.5 (0.6)	3.6 (0.9)	2.6 (1.1)
2	“I found the system unnecessarily complex.”	2.9 (1.2)	3.3 (1)	2.6 (1.7)	2.8 (1.1)
3	“I thought the system was easy to use.”	2.8 (0.7)	3 (0.8)	3 (0.7)	2.4 (0.5)
4	“I think that I would need the support of a technical person to be able to use this system.”	2.7 (1.4)	3.8 (0.5)	2.6 (1.3)	2 (1.6)
5	“I found the various functions in this system were well integrated.”	3.1 (0.8)	3 (0.8)	3.4 (0.5)	2.8 (1.1)
6	“I thought there was too much inconsistency in this system.”	3.2 (0.7)	3.3 (0.5)	3.4 (0.9)	3 (0.8)
7	“I would imagine that most people would learn to use this system very quickly.”	3.2 (0.8)	3.3 (1)	3.6 (0.5)	2.8 (0.8)
8	“I found the system very cumbersome to use.”	2.9 (1.1)	2.5 (1.3)	3.0 (1.2)	3.0 (1)
9	“I felt very confident using the system.”	2.4 (1)	2.8 (0.5)	2.8 (0.8)	1.6 (1.1)
10	“I needed to learn a lot of things before I could get going with this system.”	2.9 (1)	2.8 (1.3)	3.2 (0.8)	2.6 (1.1)

^a^Total mean 72.3 (SD 18.9).

^b^Total mean 77.5 (SD 16.2).

^c^Total mean 78 (SD 17.1).

^d^Total mean 60 (SD 22.1).

### Think Aloud Session and Final Interviews

The mean duration of the think aloud sessions in total was 45 minutes and 56 seconds (SD 5 min and 42 s; range 33 min and 34 s to 53 min and 7 s), including the instructions and the familiarization task at the beginning. The most time-consuming part was cognitive and physical testing (mean 16 min and 26 s, SD 3 min and 44 s; Table 4). Creating a test person profile was completed by all participants without any problems. Most usability problems (n=71) arose with cognitive and physical testing. The last task (“Start and finish a training with two participants simultaneously”) could not be performed by any participant (Table 4).

On the basis of the think aloud protocols, 71 different usability problems could be identified that were mentioned 134 times in total. The categorization of the usability problems according to Kushniruk and Patel [54] revealed most problems in the category, “navigation” (64/134, 47.8%), within the InCoPE-App. In particular, problems with finding the button to start a training for 2 participants simultaneously were mentioned by 79% (11/14) of the participants. The frequency of the mentioned problems and the most common examples are displayed in Table 5.

**Table 4:** Task duration and task completion.

Task	Duration, mean (SD)	Completion (N=14), n (%)		
		Success	Problem	Failure
“Create a new test person.”	3 min, 53 s (2 min, 4 s)	14 (100)	0 (0)	0 (0)
“Start and complete cognitive and physical testing with the test person.”	16 min, 26 s (3 min, 44 s)	3 (21)	10 (71)	1 (7)
“Create an exercise plan and replace two exercises.”	3 min, 54 s (1 min, 36 s)	3 (21)	4 (29)	7 (50)
“Start and finish a training session with the test person.”	5 min, 14 s (2 min, 19 s)	2 (14)	8 (57)	4 (29)
“Start and finish a training session with two participants simultaneously.”	—^a^	0 (0)	0 (0)	14 (100)

^a^Not applicable.

**Table 5:** Frequency and rating of the mentioned usability problems identified via the think aloud protocol.

Category	Mentioned frequency (N=134), n (%)	Most common problems and rating
Navigation	64 (47.8)	Finding the start button to initiate a training for 2 people—“Usability catastrophe” Changing or replacing exercises in an exercise plan—“Major usability problem” Noticing the stopwatch during assessment—“Usability catastrophe”
Screen layout	20 (14.9)	Small font type—“Major Usability Problem” Overloaded screens during exercising—“Major Usability Problem”
Graphics	6 (4.5)	No “zoom in” function—“Cosmetic problem only”
Comprehensibility	28 (20.9)	Uncertainty in cognitive test procedures—“Usability catastrophe” Unclear scientific terminology—“Major Usability Problem”
Overall usability	16 (11.9)	Drag-and-drop function is not intuitive—“Minor usability problem” Lot of information on most of the screens, owing to which app use was perceived as time consuming—“Minor usability problem”

Of the 71 identified usability concerns, 4 (6%) were rated as *usability catastrophe* according to Nielsen 48 and must be corrected before the InCoPE-App can be used in the field. Of the 71 problems, 29 (41%) were rated as a *major usability problem* with high priority to fix; 23 (32%) as *minor usability* with low priority to fix; and 8 (11%) as *cosmetic problems only*, which should only be fixed if there will be extra time for app development. Of the 71 problems, 7 (10%) mentioned usability concerns were rated as *not a usability problem at all*. Examples are displayed in Table 5.

During the final interviews, participants were able to explain which parts of the InCoPE-App were well designed. They explicitly mentioned that creating a test person within the InCoPE-App was very simple and easy to conduct:

I think, the beginning, when creating a participant profile – this was very good and clear.

Moreover, the participants highlighted the good interface and the clear user paths within the InCoPE-App:

I liked that it [the app] is well pictured.

What I totally like is that something is highlighted in orange, when I have to do [enter] something...and it is suggested to me.

The participants also liked the instructions on the training screens within the InCoPE-App:

So you’re just being carried through the exercise plan, exercise by exercise. That is well designed.

[The exercise plan] is already divided into what counts as warm-up, the workout part itself, and the cool-down. I found that to be very clear.

Overall, the participants appreciated that using the app only needs little practice and is beginner-friendly:

I generally have very little idea about a tablet or a smartphone... For me it was plausible. It [the app] has also actually indicated to me what I have to do next.

You also tried to keep it as simple as possible.

In addition to the question about the parts of the InCoPE-App they liked the most, the participants were asked to name the parts that need to be revised in their opinion. Regarding this aspect, it was mentioned that exercise videos instead of pictures would be more user-friendly:

It [the training] would take too long with the participant. I would be lost in details. Videos and especially a voice explaining it [the exercises] to me briefly, that would be very helpful for me.

This statement was accompanied by comments about information overload on the screens within the InCoPE-App:

That is a lot of text. You lose a lot of time. By the time I read this, the participants no longer have any desire

to exercise

I would have liked it better if the text had been shortened and presented in sections.

In contrast to the comments about the beginner-friendliness of the InCoPE-App, a person also mentioned barriers to the first use:

Well, if you don’t use a tablet every day, you don’t know where to push [a button]. For me as a person with limited media experience, it was hard.

Finally, when participants were asked for further comments about the InCoPE-App, they underlined that even though they had some problems with the app at first or with technologies in general, they liked the app:

At the beginning, I was really concerned. I thought that I have no idea about computer and tablets and so on. ...And I think, this is a great application, even I can handle that.

## Discussion

### Principal Findings

Promoting physical and cognitive exercise for individuals with dementia in nursing homes is critically important, particularly in terms of the reduction of PA in this setting during the COVID-19 pandemic. Nevertheless, most interventions available today have limitations regarding long-term use and implementation. With the InCoPE-App, we aimed to develop an effective and easy-to-use app that requires a multistage development process considering feedback from future end users. In this study, we analyzed the usability of the InCoPE-App, which assists nursing home staff in delivering a tailored cognitive and physical exercise program for individuals with dementia in nursing homes.

Here, we applied a mixed methods approach to get an in-depth impression of how the InCoPE-App is perceived by potential end users. Our results show that the usability of the InCoPE-App can be rated as “good” [53]. Considering the results of the single items of SUS, the least agreement was given to the statement, “I felt very confident using the system.” In contrast, the highest agreement was given to the statements, “I would imagine that most people would learn to use this system very quickly” and “I think I would like to use this system frequently.” These results indicate that on the one hand, participants felt that they needed additional information or training with the InCoPE-App. However, in contrast, they assumed that app use can be learned quickly. Overall, participants would like to use the InCoPE-App frequently and did not find the app to be unnecessarily complex.

On the basis of think aloud task completion, cognitive and physical testing required the most time. It can be assumed that this corresponds well with real-life situations, as conducting tests among individuals with dementia requires a rather large amount of time and personnel resources. We observed that, particularly, reading test instructions was time consuming. However, it is likely that time to read instructions within the InCoPE-App may decrease with more regular app use. The most difficult task (100% failure) was to start a simultaneous training of 2 individuals. This app feature needs to be revised with high priority and has to be placed more prominently within the app menu. Overall, we can assume that the InCoPE-App is a user-friendly tool and that most of the problems mentioned by participants could be solved by frequent app use.

### Comparison With Previous Studies

Although mobile devices have become increasingly popular over the past decade [28], so far, there is no scientifically evaluated mHealth app available in the context of PA promotion in nursing homes [27]. To the best of our knowledge, our study is the first to evaluate the usability of an mHealth-based app, developed to assist nursing home staff in implementing tailored cognitive and physical exercise for individuals with dementia in nursing homes. A unique feature of the InCoPE-App is that it is not used by the group considered vulnerable (ie, individuals with dementia) directly but by nursing home staff who serve as a mediator. To the best of our knowledge, there are no studies that have used this approach.

The methods used in our study are consistent with the current literature and recommendations for usability testing [37]. Both applied methods exhibit important advantages in gathering a comprehensive impression of the usability of the InCoPE-App. So far, SUS is the most frequently applied questionnaire in the usability testing of digital health solutions [31]. Although there are usability scales specially tailored for mHealth solutions (eg, mHealth Usability Questionnaire [56]), these newly developed scales have not been widely used, and only a few comparative studies exist [57]. As the sole administration of SUS as a stand-alone usability method is not recommended [58], using a think aloud protocol is a complementary approach that provides direct insight into a person’s cognitive and problem-solving processes while using an app and is therefore essential and effective for uncovering usability issues in addition to a quantitative questionnaire [37,58]. A recent systematic review showed that, even for the evaluation of usability among older participants, questionnaires and qualitative assessments such as think aloud protocols are commonly used and feasible methods [59]. Furthermore, other studies in the context of health care rehabilitation also used a mixed methods approach to assess usability [48,60,61].

In our study, we obtained a mean SUS score that is slightly above the benchmark of 68 points according to Sauro and Lewis [52] and the mean SUS for “good” usability according to Bangor et al [53]. A recent meta-analysis by Hyzy et al [62] explicitly focused on the SUS sum scores of 114 digital health apps and reported a mean score of 76.16 (SD 15.12) for all the included apps. By further categorizing the included apps, they observed a mean SUS score of 83.28 (SD 12.39) for “physical activity” apps (n=66) and a mean SUS score of 71.3 (SD 12.72) for “health care” apps [62]. Owing to the unique content of the InCoPE-App, the content-related results of the think aloud protocols and task completion are not comparable with other studies. Nevertheless, a study by Ehrler et al [48], which examined a mobile app for nurses in a hospital setting, identified “navigation within an app” to be one of the major problems. This is consistent with our results, as 47.8% (64/134) of the problems mentioned by study participants were related to the navigation structure within the InCoPE-App. These results imply that mobile apps to be used by staff in health care settings should be intuitive to navigate because complex navigation is perceived as time consuming and may thus be a barrier for long-term use by the end users [63]. Nevertheless, as the usability results of our study can be interpreted as “good,” we assume that the InCoPE-App is well designed and suitable for its primary target group, that is, nursing home staff.

The perceived usability of the InCoPE-App could also be related to the mean age and the experience with mobile apps in our sample, that is, participants aged <60 years had fewer problems with using the InCoPE-App when compared with those aged >60 years. This was also observed in another study, where older participants reported more usability problems than younger ones, who were also more likely to have used apps before study participation [48]. Furthermore, existing literature has already demonstrated generational differences and a high likelihood of problems when implementing digital (health) solutions among older adults [33,64]. Thus, an age-based digital divide in mHealth adoption has been proposed in the literature [65]. Moreover, individuals often experience a loss in digital literacy if and when they do not use digital devices on a regular basis [28]. To overcome possible age-related and experience-related barriers to app use, current literature recommends education and familiarization training [48,66]. Moreover, as the fear of making mistakes could also be perceived as a barrier [28], “undo” functions should be included in an app [48].

### Strengths and Limitations

The main strength of our study is the novelty of the presented the InCoPE-App and its user-centered development and testing process. This helps to gain new insights into a, thus far, little-explored research field. Although our participants were predominantly women and aged >50 years, they can be considered to be representative of the population of end users (ie, nursing home staff) who will use the system in the future. It is very crucial to include a representative target group to generate valid usability data and to avoid biases [37]. In addition, our sample was heterogeneous in terms of age, education, and technical experience. This allowed us to detect usability problems from different perspectives and gave us a nuanced impression of the potential end users. Moreover, engaging individuals with less access to or knowledge about technology is very important to ensure high usability of a system for individuals with low digital literacy [67]. Another strength of the study is the mixed methods approach. Particularly in usability research, 1 method alone is not suitable to cover all the important aspects of a system’s usability. Combining SUS with the think aloud task and the interview questions therefore allowed us to gain deep insight into the usability problems, as opposed to only evaluating usability on the basis of a sum score.

A limitation of our study is the relatively late inclusion of the end users in the direct development process of the InCoPE-App. Although we created fictitious end users on the basis of a questionnaire in early development stages [26], the main content and the basic structure of the data model has been developed and finalized without the input of nursing home staff. In other studies, end users were included from the very beginning of the app development process [60]. It is likely that some of the frequently mentioned usability problems (eg, navigation within the app) could have been avoided by the early inclusion of end users in the development process. Another limitation is that members of our research team ranked the usability problems according to the method of Nielsen [55], and it is possible that the end users would have rated the severity of the problems differently. Thus, the revision of the app based on the prioritization done by the researchers may not fully correspond to the expectations and wishes of the end users as they may have chosen another prioritization. Therefore, in future studies, end users should also be included in this step. Furthermore, it should be differentiated which usability problems should be further addressed from different perspectives (eg, experts, developers, researchers, and end users).

### Conclusions

The InCoPE-App is a novel and innovative app that assists nursing home staff in delivering tailored cognitive and physical exercise to individuals with dementia residing in nursing homes. We showed that the usability of the current version of the InCoPE-App can be rated as good according to 14 potential end users. Furthermore, even older participants found the InCoPE-App as easy to use after some familiarization. Nevertheless, certain aspects such as navigation features within the app must be further improved to increase the usability of the app in the future. To overcome potential barriers to using the app, further development should follow a “less is more” approach, for example, by minimizing navigation screens or reducing the complexity and length of text on the screens. Overall, the inclusion of end users in the app’s development process continues to be critically relevant and highly important. Therefore, the InCoPE-App was further tested in an 18-week intervention study [68].

## Appendix

Multimedia Appendix 1 Main functions of the Individualized Cognitive and Physical Exercise-App.

## Abbreviations

the InCoPE-App: the Individualized Cognitive and Physical Exercise-App
mHealth: mobile health
PA: physical activity
QoL: quality of life
SUS: System Usability Scale

## References

[1] (2023). Dementia: key facts. World Health Organization.

[2] Patterson C (2018). World Alzheimer report 2018 - the state of the art of dementia research: new frontiers. Alzheimer's Disease International.

[3] Pickett J, Bird C, Ballard C, Banerjee S, Brayne C, Cowan K, Clare L, Comas-Herrera A, Corner L, Daley S, Knapp M, Lafortune L, Livingston G, Manthorpe J, Marchant N, Moriarty J, Robinson L, van Lynden C, Windle G, Woods B, Gray K, Walton C (2018). A roadmap to advance dementia research in prevention, diagnosis, intervention, and care by 2025. Int J Geriatr Psychiatry.

[4] Forbes D, Forbes SC, Blake CM, Thiessen EJ, Forbes S (2015). Exercise programs for people with dementia. Cochrane Database Syst Rev.

[5] Lam FM, Huang MZ, Liao LR, Chung RC, Kwok TC, Pang MY (2018). Physical exercise improves strength, balance, mobility, and endurance in people with cognitive impairment and dementia: a systematic review. J Physiother.

[6] Ojagbemi A, Akin-Ojagbemi N (2019). Exercise and quality of life in dementia: a systematic review and meta-analysis of randomized controlled trials. J Appl Gerontol.

[7] Müllers P, Taubert M, Müller NG (2019). Physical exercise as personalized medicine for dementia prevention?. Front Physiol.

[8] Barisch-Fritz B, Trautwein S, Scharpf A, Krell-Roesch J, Woll A (2022). Effects of a 16-week multimodal exercise program on physical performance in individuals with dementia: a multicenter randomized controlled trial. J Geriatr Phys Ther.

[9] Bezold J, Trautwein S, Barisch-Fritz B, Scharpf A, Krell-Roesch J, Nigg CR, Woll A (2021). Effects of a 16-week multimodal exercise program on activities of daily living in institutionalized individuals with dementia. Ger J Exerc Sport Res.

[10] Trautwein S, Barisch-Fritz B, Scharpf A, Ringhof S, Stein T, Krell-Roesch J, Woll A (2020). Effects of a 16-week multimodal exercise program on gait performance in individuals with dementia: a multicenter randomized controlled trial. BMC Geriatr.

[11] Hoffmann F, Kaduszkiewicz H, Glaeske G, van den Bussche H, Koller D (2014). Prevalence of dementia in nursing home and community-dwelling older adults in Germany. Aging Clin Exp Res.

[12] Auer SR, Höfler M, Linsmayer E, Beránková A, Prieschl D, Ratajczak P, Šteffl M, Holmerová I (2018). Cross-sectional study of prevalence of dementia, behavioural symptoms, mobility, pain and other health parameters in nursing homes in Austria and the Czech Republic: results from the DEMDATA project. BMC Geriatr.

[13] Schäufele M, Köhler L, Hendlmeier I, Hoell A, Weyerer S (2013). Prevalence of dementia and medical care in German nursing homes: a nationally representative survey. Psychiatr Prax.

[14] Kowalska J, Rymaszewska J, Szczepańska-Gieracha J (2013). Occurrence of cognitive impairment and depressive symptoms among the elderly in a nursing home facility. Adv Clin Exp Med.

[15] Seitz D, Purandare N, Conn D (2010). Prevalence of psychiatric disorders among older adults in long-term care homes: a systematic review. Int Psychogeriatr.

[16] Brent RJ (2022). Life expectancy in nursing homes. Appl Econ.

[17] Król-Zielińska M, Kusy K, Zieliński J, Osiński W (2011). Physical activity and functional fitness in institutionalized vs. independently living elderly: a comparison of 70-80-year-old city-dwellers. Arch Gerontol Geriatr.

[18] Olsen C, Pedersen I, Bergland A, Enders-Slegers MJ, Jøranson N, Calogiuri G, Ihlebæk C (2016). Differences in quality of life in home-dwelling persons and nursing home residents with dementia - a cross-sectional study. BMC Geriatr.

[19] Frahsa A, Altmeier D, John JM, Gropper H, Granz H, Pomiersky R, Haigis D, Eschweiler GW, Nieß AM, Sudeck G, Thiel A (2020). "I trust in staff's creativity"-the impact of COVID-19 lockdowns on physical activity promotion in nursing homes through the lenses of organizational sociology. Front Sports Act Living.

[20] Geissler T, Bezold J, Scharpf A, Barisch-Fritz B, Trautwein S, Krell-Roesch J, Woll A (2021). Impact of lockdown restrictions on physical activity programs in nursing homes during the COVID-19 pandemic. Proceedings of the 26th Annual Congress of the European College of Sport Science.

[21] Kaelen S, van den Boogaard W, Pellecchia U, Spiers S, De Cramer C, Demaegd G, Fouqueray E, Van den Bergh R, Goublomme S, Decroo T, Quinet M, Van Hoof E, Draguez B (2021). How to bring residents' psychosocial well-being to the heart of the fight against COVID-19 in Belgian nursing homes-a qualitative study. PLoS One.

[22] Istepanian R, Jovanov E, Zhang YT (2004). Introduction to the special section on M-Health: beyond seamless mobility and global wireless health-care connectivity. IEEE Trans Inf Technol Biomed.

[23] Nacinovich M (2011). Defining mHealth. J Commun Healthc.

[24] (2011). mHealth: new horizons for health through mobile technologies: second global survey on eHealth. World Health Organization.

[25] Bhattacharya S, Kumar A, Kaushal V, Singh A (2018). Applications of m-health and e-health in public health sector: the challenges and opportunities. Int J Med Public Health.

[26] Barisch-Fritz B, Barisch M, Trautwein S, Scharpf A, Bezold J, Woll A (2019). Designing a mobile app for treating individuals with dementia: combining UX research with sports science. Proceedings of the 12th International Symposium on Computer Science in Sport.

[27] Diener J, Rayling S, Bezold J, Krell-Roesch J, Woll A, Wunsch K (2022). Effectiveness and acceptability of e- and m-health interventions to promote physical activity and prevent falls in nursing homes-a systematic review. Front Physiol.

[28] Odendaal WA, Anstey Watkins J, Leon N, Goudge J, Griffiths F, Tomlinson M, Daniels K (2020). Health workers' perceptions and experiences of using mHealth technologies to deliver primary healthcare services: a qualitative evidence synthesis. Cochrane Database Syst Rev.

[29] Shackel B (2009). Usability – context, framework, definition, design and evaluation. Interact Comput.

[30] Thirumalai M, Rimmer JH, Johnson G, Wilroy J, Young HJ, Mehta T, Lai B (2018). TEAMS (Tele-Exercise and Multiple Sclerosis), a tailored telerehabilitation mHealth app: participant-centered development and usability study. JMIR Mhealth Uhealth.

[31] Maramba I, Chatterjee A, Newman C (2019). Methods of usability testing in the development of eHealth applications: a scoping review. Int J Med Inform.

[32] Nouri R, R Niakan Kalhori S, Ghazisaeedi M, Marchand G, Yasini M (2018). Criteria for assessing the quality of mHealth apps: a systematic review. J Am Med Inform Assoc.

[33] Guo C, Ashrafian H, Ghafur S, Fontana G, Gardner C, Prime M (2020). Challenges for the evaluation of digital health solutions-a call for innovative evidence generation approaches. NPJ Digit Med.

[34] Zapata BC, Fernández-Alemán JL, Idri A, Toval A (2015). Empirical studies on usability of mHealth apps: a systematic literature review. J Med Syst.

[35] Tsertsidis A (2021). Identifying digital solutions for people with dementia (PwD): lessons learned from a Swedish dementia care residence. Gerontechnology.

[36] Cho H, Yen PY, Dowding D, Merrill JA, Schnall R (2018). A multi-level usability evaluation of mobile health applications: a case study. J Biomed Inform.

[37] Jaspers MW (2009). A comparison of usability methods for testing interactive health technologies: methodological aspects and empirical evidence. Int J Med Inform.

[38] Folstein MF, Folstein SE, McHugh PR (1975). "Mini-mental state". A practical method for grading the cognitive state of patients for the clinician. J Psychiatr Res.

[39] Rossiter-Fornoff JE, Wolf SL, Wolfson LI, Buchner DM (1995). A cross-sectional validation study of the FICSIT common data base static balance measures. Frailty and injuries: cooperative studies of intervention techniques. J Gerontol A Biol Sci Med Sci.

[40] Graham JE, Ostir GV, Kuo YF, Fisher SR, Ottenbacher KJ (2008). Relationship between test methodology and mean velocity in timed walk tests: a review. Arch Phys Med Rehabil.

[41] Blankevoort CG, van Heuvelen MJ, Scherder EJA (2013). Reliability of six physical performance tests in older people with dementia. Phys Ther.

[42] Jones CJ, Rikli RE, Beam WC (1999). A 30-s chair-stand test as a measure of lower body strength in community-residing older adults. Res Q Exerc Sport.

[43] Bossers WJ, van der Woude LH, Boersma F, Scherder EJ, van Heuvelen MJ (2012). Recommended measures for the assessment of cognitive and physical performance in older patients with dementia: a systematic review. Dement Geriatr Cogn Dis Extra.

[44] Trautwein S, Barisch-Fritz B, Scharpf A, Bossers W, Meinzer M, Steib S, Stein T, Bös K, Stahn A, Niessner C, Altmann S, Wittelsberger R, Woll A (2019). Recommendations for assessing motor performance in individuals with dementia: suggestions of an expert panel - a qualitative approach. Eur Rev Aging Phys Act.

[45] Barisch-Fritz B, Bezold J, Scharpf A, Trautwein S, Krell-Roesch J, Woll A (2022). Usability and effectiveness of an individualized, tablet-based, multidomain exercise program for people with dementia delivered by nursing assistants: protocol for an evaluation of the InCoPE-app. JMIR Res Protoc.

[46] Barisch-Fritz B, Bezold J, Trautwein S, Scharpf A, Woll A (2019). Cluster analysis of motor and cognitive skills of institutionalized individuals with dementia: 4 phenotypes for developing individualized physical activity programs. Proceedings of the 24th Annual Congress of the European College of Sports Science.

[47] Barisch-Fritz B, Bezold J, Barisch M, Scharpf A, Geissler T, Trautwein S, Krell-Rösch J, Woll A, Baca A, Exel J, Lames M, James N, Parmar N (2022). InCoPE-App: a digital solution to train individuals with dementia. Usability review based on expert user experiences. Proceedings of the 9th International Performance Analysis Workshop and Conference & 5th IACSS Conference.

[48] Ehrler F, Weinhold T, Joe J, Lovis C, Blondon K (2018). A mobile app (BEDSide Mobility) to support nurses' tasks at the patient's bedside: usability study. JMIR Mhealth Uhealth.

[49] Brooke J, Jordan PW, Thomas B, McClelland IL, Weerdmeester B (1996). SUS: a "quick and dirty" usability scale. Usability Evaluation in Industry.

[50] Gao M, Kortum P, Oswald FL (2020). Multi-language toolkit for the system usability scale. Int J Hum Comput Interact.

[51] Lewis JR (2018). The system usability scale: past, present, and future. Int J Hum Comput.

[52] Sauro J, Lewis JR (2016). Quantifying the User Experience: Practical Statistics for User Research. 2nd edition.

[53] Bangor A, Kortum P, Miller J (2009). Determining what individual SUS scores mean: adding an adjective rating scale. J Usability Stud.

[54] Kushniruk AW, Patel VL (2004). Cognitive and usability engineering methods for the evaluation of clinical information systems. J Biomed Inform.

[55] Nielsen J (2010). Usability Engineering.

[56] Zhou L, Bao J, Setiawan IM, Saptono A, Parmanto B (2019). The mHealth app usability questionnaire (MAUQ): development and validation study. JMIR Mhealth Uhealth.

[57] Hajesmaeel-Gohari S, Khordastan F, Fatehi F, Samzadeh H, Bahaadinbeigy K (2022). The most used questionnaires for evaluating satisfaction, usability, acceptance, and quality outcomes of mobile health. BMC Med Inform Decis Mak.

[58] Broekhuis M, van Velsen L, Hermens H (2019). Assessing usability of eHealth technology: a comparison of usability benchmarking instruments. Int J Med Inform.

[59] Wang Q, Liu J, Zhou L, Tian J, Chen X, Zhang W, Wang H, Zhou W, Gao Y (2022). Usability evaluation of mHealth apps for elderly individuals: a scoping review. BMC Med Inform Decis Mak.

[60] Rai HK, Schneider J, Orrell M (2020). An individual cognitive stimulation therapy app for people with dementia: development and usability study of thinkability. JMIR Aging.

[61] Reeder B, Drake C, Ozkaynak M, Wald HL (2019). Usability testing of a mobile clinical decision support app for urinary tract infection diagnosis in nursing homes. J Gerontol Nurs.

[62] Hyzy M, Bond R, Mulvenna M, Bai L, Dix A, Leigh S, Hunt S (2022). System usability scale benchmarking for digital health apps: meta-analysis. JMIR Mhealth Uhealth.

[63] Ahmad NA, Mat Ludin AF, Shahar S, Mohd Noah SA, Mohd Tohit N (2022). Willingness, perceived barriers and motivators in adopting mobile applications for health-related interventions among older adults: a scoping review. BMJ Open.

[64] Calvo-Porral C, Pesqueira-Sanchez R (2020). Generational differences in technology behaviour: comparing millennials and generation X. Kybernetik.

[65] Fox G, Connolly R (2018). Mobile health technology adoption across generations: narrowing the digital divide. Inf Syst J.

[66] Mayer MA, Rodríguez Blanco O, Torrejon A (2019). Use of health apps by nurses for professional purposes: web-based survey study. JMIR Mhealth Uhealth.

[67] Richardson J, Letts L, Sinclair S, Chan D, Miller J, Donnelly C, Smith-Turchyn J, Wojkowski S, Gravesande J, Loyola Sánchez A (2021). Using a web-based app to deliver rehabilitation strategies to persons with chronic conditions: development and usability study. JMIR Rehabil Assist Technol.

[68] Barisch-Fritz B, Bezold J, Scharpf A, Trautwein S, Krell-Roesch J, Woll A (2022). ICT-based individualized training of institutionalized individuals with dementia. Evaluation of usability and trends toward the effectiveness of the InCoPE-app. Front Physiol.

