# Supplementary material for: A Tablet-Based App to Support Nursing Home Staff in Delivering an Individualized Cognitive and Physical Exercise Program for Individuals With Dementia: Mixed Methods Usability Study
Source: JMIR Aging. 2023 Aug 22;6:e46480. doi: 10.2196/46480 (PMC10481225; doi:10.2196/46480)
Supplement: Multimedia Appendix 1 [file aging_v6i1e46480_app1.docx]

## Supplementary file

Supplementary Table 1: Main functions of the InCoPE-App

| Function | Description |
| --- | --- |
|  |  |
| 1. Main menu | The main menu allows the user to navigate between all app functions. |
| 1. Exercise pool | The exercise pool shows and describes each exercise integrated in the app and with various degrees of difficulty. This function also allows access to single exercises without starting an entire training program with an individual participant. |
| 1. Test pool | The test pool presents each cognitive or physical performance assessment integrated in the app. This function also allows access to single tests without starting the entire test battery. |
| 1. Participant overview | The required action/ next steps (i.e., test, training) is displayed for each partici-pant. |
| 1. Participant’s details | The personal information of a chosen participant is shown (e.g., age, height, type of dementia). Furthermore, serious events (e.g., illness) can be tracked. |
| 1. Performing test battery | This function allows to assess and record the current cognitive and physical performance of a participant. A test battery of cognitive and physical perfor-mance tests is integrated. Each test and its recorded measurements/data (e.g., required time, repetitions) are described. Furthermore, results can be entered directly. The results are used to automatically create an individualized exercise training plan for each participant. |
| 1. Training schedule preview | The training schedule preview allows the user to take a look at the exercise training plan before starting the training. Furthermore, the equipment needed for the exercises is listed. |
| 1. Training | This function leads a user through an entire training session with a participant. Each exercise is described in detail, including written descriptions, pictures, and common mistakes or possible risks. At the end of each training session, the training can be rated from 0 to five stars. |
